# A novel risk score to predict cardiovascular complications in patients with coronavirus disease 2019 (COVID‐19): A retrospective, multicenter, observational study

**DOI:** 10.1002/iid3.353

**Published:** 2020-09-24

**Authors:** Dong Huang, Huan Yang, He Yu, Ting Wang, Rong Yao, Zongan Liang

**Affiliations:** ^1^ Department of Respiratory and Critical Care Medicine, West China Hospital Sichuan University Chengdu Sichuan China; ^2^ Emergency Medical Laboratory, Department of Emergency Medicine, West China Hospital Sichuan University Chengdu Sichuan China; ^3^ Disaster Medical Center Sichuan University Chengdu Sichuan China

**Keywords:** cardiovascular complications, clinical feature, COVID‐19, predictive risk score

## Abstract

**Background:**

We conducted this study to explore a novel risk score to predict cardiovascular complications in patients with coronavirus disease 2019 (COVID‐19).

**Methods:**

The current study was a retrospective, multicenter, observational study. The clinical data of COVID‐19 patients at admission were collected. Patients were randomly divided into training set and testing set (70% vs. 30% of patients). Independent risk factors were identified via logistic regression analysis.

**Results:**

Finally, 1207 patients were included. Ten independent risk factors associated with cardiovascular complications were identified in training set: male (odds ratio [OR]: 1.84; 95% confidence interval [CI]: 1.18, 2.85), age ≥ 60 years old (OR: 2.01; 95% CI: 1.3, 3.2), cough (OR: 1.86; 95% CI: 1.16, 3), chronic heart disease (OR: 2.3; 95% CI: 1.19, 4.46), lymphocyte count ≤1.1 × 10^9^/L at admission (OR: 1.60; 95% CI: 1.03, 2.47), blood urea nitrogen ≥7 mmol/L at admission (OR: 2.14; 95% CI: 1.27, 3.62), estimated glomerular filtration rate ≤90 ml/min/1.73 m^2^ at admission (OR: 2.08; 95% CI: 1.13, 3.83), activated partial thromboplastin time ≥37 s (OR: 3.07; 95% CI: 1.37, 6.86), D‐dimer ≥ 0.5 mg/L (OR: 2.12; 95% CI: 1.33, 3.36) and procalcitonin ≥0.5 μg/L (OR: 3.58; 95% CI: 1.40, 9.14). The area under curve of ROC curve was 0.773 (95% CI: 0.723, 0.822; *p* < .01). The risk score had robustness and generalizability after validation. Cardiovascular complications were significantly associated with poorer survivals (log‐rank test: *p* < .001).

**Conclusions:**

We developed and validated a novel risk score, which has a promising predictive capacity for cardiovascular complications in COVID‐19 patients.

## Graphical abstract

We developed and validated a novel risk score, which has a promising predictive capacity for cardiovascular complications in coronavirus disease 2019 (COVID‐19) patients. Cardiovascular complications were significantly associated with poorer survival (log‐rank test: *p* < .001).

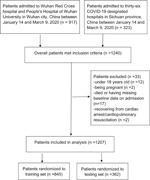

## 1 BACKGROUND

The outbreak of novel coronavirus disease 2019 (COVID‐19), caused by severe acute respiratory syndrome coronavirus 2 (SARS‐CoV‐2), began in December 2019.^1^, ^2^ It has led to a pandemic which caused over 17 million infected people and 600,000 deaths in over 200 countries/regions so far.^3^ This new pandemic has also created an unprecedented burden on healthcare systems throughout the world, highlighting the urgency to improve the hospital management and early identification and stratification of patients.

SARS‐CoV‐2 is considered principally as a respiratory pathogen and the respiratory symptoms are the most common symptoms. However, extrapulmonary manifestations or injury should not be ignored as well. It has been reported that pre‐existing cardiovascular diseases are associated with worse prognosis in COVID‐19 patients.^4^ In contrast, cardiovascular complications caused by COVID‐19, including arrhythmias, myocardial infarction (MI), myocarditis, and heart failure (HF), and so forth are also significant contributors to increased mortality and rate of admission to intensive care unit (ICU) of COVID‐19 patients.^4^, ^5^, ^6^, ^7^, ^8^, ^9^ Therefore, it is of the greatest importance to explore the damage caused by the SARS‐CoV‐2 to the cardiovascular system.

Meanwhile, it is also of considerable value to identify risk factors for predicting potential cardiovascular complications at an early stage, which could guide and optimize the therapies for COVID‐19 under the circumstance of limited healthcare resources. However, related studies are still insufficient up to now. Early prediction models that combine clinical features to identify COVID‐19 patients at high risk of cardiovascular events remain poorly defined and challenging to investigate. The current retrospective, multicenter, observational study was conducted aiming to develop and validate a novel risk score used for predicting cardiovascular complications, and also to assess the relationship between these complications and prognosis among COVID‐19 patients.

## 2 MATERIALS AND METHODS

### 2.1 Study design

This retrospective, multicenter, observational study of laboratory‐confirmed COVID‐19 patients was conducted in accordance with the amended Declaration of Helsinki and approved by the West China Hospital of Sichuan University Biomedical Research Ethics Committee (No. 2020‐272). Written informed consent was waived because of the urgent need to collect clinical data and retrospective observational design.

Clinical data of hospitalized patients from two major COVID‐19 designated hospitals (Wuhan Red Cross hospital and People's Hospital of Wuhan University) in Wuhan city, and 36 COVID‐19‐designated hospitals in Sichuan province, China between January 14 and March 9, 2020 were collected and analyzed. All included patients were randomly divided into training set and testing set (70% percent vs. 30% of patients). The training set was used to develop a risk score and a testing set was applied to validate the robustness and generalizability of the risk score.

All patient data were anonymously recorded to ensure confidentiality. Two doctors reviewed the medical records of all patients independently. Any disagreement was resolved through the third doctor and team discussion until consensus reached.

### 2.2 Participants and data collection

All patients enrolled in this study were diagnosed with confirmed COVID‐19 according to World Health Organization interim guidance.^10^ The confirmed case is defined as positive result of the nucleic acid of SARS‐CoV‐2 by real‐time reverse‐transcription polymerase chain reaction.

The following exclusion criteria were used: (1) under 18 years old; (2) being pregnant; (3) died or having missing baseline data on admission; (4) recovering from cardiac arrest/cardiopulmonary resuscitation.

Demographic characteristics, basic vital signs, symptoms and signs, comorbidities, chest computed tomography (CT) scan images, and laboratory examinations data were retrospectively collected from electronic medical records. All these baseline data were recorded at admission or within 24 h after admission to hospitals. Continuous variables were categorized for further analysis. The threshold value of each continuous variable was determined by the clinically relevant cut‐off value, or upper limit or lower limit of the normal range. Two doctors completed the data collection independently.

### 2.3 Outcomes

The occurrence of cardiovascular complications was considered if any of the following appeared during hospitalization: (1) acute myocardial injury; (2) acute myocardial infarction (AMI), including non‐ST elevation or ST elevation MI; (3) new or worsening HF; (4) de novo arrhythmia; (5) deep vein thrombosis (DVT) or pulmonary embolism (PE).

This composite endpoint has been used in previous studies that evaluated the cardiovascular complications of pneumonia.^11^, ^12^ According to Fourth Universal Definition of Myocardial Infarction,^13^ myocardial injury was diagnosed by the detection of elevated cardiac troponin with at least 1 value above the 99th percentile upper reference limit, and criteria for AMI were acute myocardial injury with at least one of the following: symptoms of myocardial ischemia; new ischemic electrocardiograph (ECG) changes; imaging evidence of new loss of viable myocardium or new regional wall motion abnormality; identification of a coronary thrombus by angiography or autopsy. New or worsening HF was considered in patients with clinical signs (such as pulmonary edema, acute congestive HF, cardiomegaly, vascular congestion, etc.) and supportive findings on ECG or chest radiograph.^14^ De novo arrhythmia was determined on the basis of a new episode of arrhythmia documented by ECGs during hospitalization, which was not detected before hospital admission. DVT or PE was considered on the basis of clinical manifestations and supportive findings of ultrasound or angiography CT.

Likewise, two doctors reviewed and checked the diagnosis of cardiovascular complications independently. Any disagreement was resolved through the third doctor and team discussion until consensus reached.

### 2.4 Statistical analysis

Data were analyzed by using IBM SPSS Statistics version 23.0 (SPSS). Data were expressed as mean ± standard deviation or median (interquartile range) for continuous variables, as well as counts and percentages for categorical variables. The data were tested by the Kolmogorov–Smirnov normality test and Bartlett's test for homogeneity of variance. The difference between the two groups was tested using a two‐tailed independent Student's *t* tests for normally distributed continuous variables, the Mann–Whitney *U* test for non‐normally distributed continuous variables, and *χ* ^2^ test or Fisher's exact test for categorical variables.

Variables with *p* < .10 were included in univariate and multivariate logistic regression analysis to identify independent risk factors. The odds ratio (OR) and confidence interval (CI) were used to evaluate risk factors. The score for each independent risk factor was assigned as an integer value close to the regression coefficient. The total risk score of each patient is the sum of each single score. To assess the accuracy of risk score as a predictor of cardiovascular complications of COVID‐19, the receiver operating characteristics (ROC) curve was conducted and the area under the ROC curve (AUC) was reported. The optimal cut‐off point of the risk score was based on the Youden's index of ROC curve, corresponding to the maximum joint sensitivity and specificity.

After that, the variables required for calculating the risk score were collected, calculated, and tested for the testing set. The performance of risk score in training set, testing set, and overall patients were compared. We also conducted survival analysis in all patients using the Kaplan–Meier method and log‐rank test to explore the impacts of cardiovascular complications on the prognosis of COVID‐19 patients. *p* < .05 was considered statistically significant.

## 3 RESULTS

### 3.1 Baseline patient characteristics

**Figure 1:**
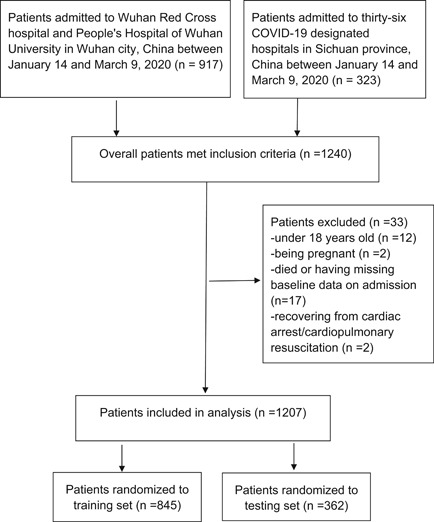
Study population. COVID‐19, coronavirus disease 2019

A total of 1240 patients confirmed with COVID‐19 were retrospectively enrolled in the study. Ultimately, 33 patients were excluded according to the exclusion criteria, and 1207 patients were analyzed. Among them, 845 patients (70% of overall patients) were randomized to training set and 362 patients (30% of overall patients) were included in the testing set (Figure 1).

In the training set, 122 (14.4%) patients were found to have cardiovascular complications. Among them, 98 patients had acute myocardial injury and 5 patients further developed AMI. A total of 8 patients had new or worsening HF and 30 patients had de novo arrhythmia. Additionally, only one patient was diagnosed with DVT. The rate of male and the median age of patients with complications were both significantly higher than those of patients without complications (63.9% vs. 44.3%, *p* < .001; 64.5 vs. 53 years old, *p* < .001). There were also some other significant differences between patients with or without cardiovascular complications in terms of fever (*p* = .031), cough (*p* = .002), weakness/fatigue (*p* = .035), unconsciousness (*p* = .036), chronic heart disease (*p* < .001), diabetes mellitus (*p* = .036).

Furthermore, some differences in laboratory examinations were also demonstrated. Compared with patients without cardiovascular complications, patients with cardiovascular complications had higher white blood cell counts (6.11 vs. 5.43 × 10^9^/L; *p* = .001), neutrophil counts (4.73 vs. 3.29 × 10^9^/L; *p* < .001), aspartate aminotransferase (30.5 vs. 23.7 U/L; *p* < .001), and so on; however, lower lymphocyte counts (0.9 vs. 1.25 × 10^9^/L; *p* < .001), platelet counts (184 vs. 207 × 10^9^/L; *p* = .005), estimated glomerular filtration rate (eGFR; 85.8 vs. 102.3 ml/min/1.73 m^2^; *p* < .001), and so forth. The detailed baseline characteristics of the patients are shown in Table 1.

**Table 1:** Comparisons of clinical characteristics between patients with cardiovascular complications and patients without cardiovascular complications in the training set

Variables	Overall (*n* = 845)	With cardiovascular complications (*n* = 122)	Without cardiovascular complications (*n* = 723)	*p* value
*Demographic characteristics*				
Sex (male)	398 (47.1)	78 (63.9)	320 (44.3)	<.001
Age (years)	55 (40, 65)	64.5 (47, 73)	53 (39, 63)	<.001
≥60	333 (39.4)	76 (62.3)	257 (35.5)	<.001
History of alcohol use	196 (23.3)	29 (23.8)	167 (23.1)	.871
Smoking history	233 (27.6)	41 (33.6)	192 (26.6)	.107
*Vital signs on admission*				
Temperature (°C)	36.7 (36.4, 37.1)	36.7 (36.5, 37.5)	36.7 (36.4, 37)	.052
Respiratory rate (breath/min)	20 (19, 21)	20 (19, 23)	20 (19, 21)	.113
Heart rate (beat/min)	85 (78, 96)	85 (78, 96)	86 (78, 96)	.755
Systolic pressure (mmHg)	128 (118, 140)	129 (118, 140)	128 (118, 140)	.515
Diastolic pressure (mmHg)	80 (71, 86)	80 (73, 88)	79 (71, 86)	.262
Glasgow Coma Scale	15 (15, 15)	15 (15, 15)	15 (15, 15)	.726
*Symptoms and signs*				
Fever	558 (66)	91 (74.6)	467 (64.6)	.031
Cough	525 (62.1)	91 (74.6)	434 (60)	.002
Hemoptysis	30 (3.6)	5 (4.1)	25 (3.5)	.724
Shortness of breath/dyspnea	190 (22.5)	35 (28.7)	155 (21.4)	.076
Weakness/fatigue	309 (36.6)	55 (45.1)	254 (35.1)	.035
Sore throat	71 (8.4)	11 (9)	60 (8.3)	.792
Rhinorrhea	22 (2.6)	1 (0.8)	21 (2.9)	.303
Earache/ear pain	10 (1.2)	1 (0.8)	9 (1.2)	1
Wheeze	99 (11.7)	19 (15.6)	80 (11.1)	.152
Stuffy nose	16 (1.9)	0 (0)	16 (2.2)	.194
Chest pain/distress	192 (22.7)	30 (24.6)	162 (22.4)	.594
Muscle ache/myalgia	72 (8.5)	11 (9)	61 (8.4)	.832
Arthralgia	13 (1.5)	4 (3.3)	9 (1.2)	.197
Headache	45 (5.3)	9 (7.4)	36 (5)	.275
Unconsciousness	9 (1.1)	4 (3.3)	5 (0.7)	.036
Stomachache	12 (1.4)	2 (1.6)	10 (1.4）	1.00
Nausea/vomiting	29 (3.4)	2 (1.6)	27 (3.7)	.364
Diarrhea	95 (11.2)	11 (9)	84 (11.6)	.4
*Comorbidities*				
COPD	22 (2.6)	5 (4.1)	17 (2.4)	.416
Asthma	6 (0.7)	0 (0)	6 (0.8)	.601
Hypertension	225 (26.6)	40 (32.8)	185 (25.6)	.096
Chronic heart disease	61 (7.2)	20 (16.4)	41 (5.7)	<.001
Chronic liver disease	65 (7.7)	10 (8.2)	55 (7.6)	.821
Diabetes mellitus	127 (15)	26 (21.3)	101 (14)	.036
Cancer	20 (2.4)	2 (1.6)	18 (2.5)	.803
Cerebrovascular disease	14 (1.7)	2 (1.6)	12 (1.7)	1.00
Chronic kidney disease	14 (1.7)	4 (3.3)	10 (1.4)	.257
*Chest CT scan images*				
Abnormal chest image	566 (67)	74 (60.7)	492 (68)	.108
Ground‐glass opacity	531 (62.8)	71 (58.2)	460 (63.6)	.251
Presence with consolidation	323 (38.2)	49 (40.2)	274 (37.9)	.643
*Laboratory examinations*				
White blood cell count (×10^9^/L)	5.5 (4.25, 7.19)	6.11 (4.41, 8.27)	5.43 (4.2, 6.99)	.001
≥9.5	76 (9)	22 (18)	54 (7.5)	<.001
Neutrophils count (×10^9^/L)	3.43 (2.42, 5.0)	4.73 (2.67, 6.99)	3.29 (2.36, 4.65)	<.001
≥6.3	109 (12.9)	34 (27.9)	75 (10.4)	<.001
Lymphocyte count (×10^9^/L)	1.2 (0.84, 1.70)	0.9 (0.59, 1.29)	1.25 (0.9, 1.7)	<.001
≤1.1	339 (40.1)	74 (60.7)	265 (36.7)	<.001
Monocyte count (×10^9^/L)	0.43 (0.30, 0.58)	0.41 (0.25, 0.58)	0.44 (0.3, 0.58)	.27
Hemoglobin (g/L)	128 (117, 140)	129.5 (119, 142)	128 (117, 140)	.344
Hematocrit	0.39 (0.34, 4.31)	0.39 (0.35, 36.7)	0.38 (0.34, 0.52)	.395
Erythrocyte sedimentation rate	31 (18, 55)	32 (15, 73)	31 (18, 52)	.401
Platelet count (×10^9^/L)	201 (154, 269)	184 (136, 242)	207 (157, 271)	.005
≤100	41 (4.9)	11 (9)	30 (4.1)	.02
Triglyceride	1.24 (0.94, 1.79)	1.32 (1.03, 2.02)	1.24 (0.93, 1.76)	.161
Cholesterol	4.04 (3.44, 4.74)	4.02 (3.38, 4.56)	4.05 (3.48, 4.78)	.482
High‐density lipoprotein	1.03 (0.84, 1.29)	1 (0.79, 1.3)	1.03 (0.86, 1.29)	.145
Low‐density lipoprotein	2.44 (1.94, 2.99)	2.38 (1.95, 2.75)	2.45 (1.94, 3.07)	.19
ALT (U/L)	23.3 (15.2, 39)	27 (15.6, 45)	23 (15.1, 38)	.193
AST (U/L)	24 (19, 34)	30.5 (22.8, 46.3)	23.7 (19, 33)	<.001
≥35	174 (20.6)	42 (34.4)	132 (18.3)	<.001
Total bilirubin (μmol/L)	10.2 (7.5, 14.5)	11.8 (8.6, 16.2)	10 (7.3, 13.9)	.002
≥17.1	106 (12.5)	21 (17.2)	85 (11.8)	.09
Direct bilirubin (μmol/L)	3.3 (2.2, 4.8)	4.3 (2.6, 6.2)	3.2 (2.2, 4.5)	<.001
≥6.8	90 (10.7)	24 (19.7)	66 (9.1)	<.001
Indirect bilirubin	7 (4.9, 9.9)	7.7 (5.1, 10.4)	6.8 (4.8, 9.7)	.266
Blood urea nitrogen (mmol/L)	4.38 (3.4, 5.73)	5.4 (3.9, 7.4)	4.2 (3.4, 5.5)	<.001
≥7	116 (13.7)	38 (31.1)	78 (10.8)	<.001
Creatinine (μmol/L)	61 (50, 73)	69 (57, 83)	60 (49, 72)	<.001
≥106	28 (3.3)	11 (9)	17 (2.4)	<.001
eGFR (ml/min/1.73 m^2^)	100.9 (89, 114.7)	85.8 (62.3, 102.7)	102.3 (91.7, 117.3)	<.001
≤90	79 (9.3)	25 (20.5)	54 (7.5)	<.001
Uric acid	257 (204, 334)	264.5 (202, 362)	255 (205, 332)	.269
Total protein	64.4 (59.8, 69.2)	62.5 (57.3, 67.7)	65 (60.3, 69.3)	.007
≤60	210 (24.9)	49 (40.2)	161 (22.3)	<.001
Albumin (g/L)	39 (34.8, 42.8)	35.8 (31.9, 40.2)	39.6 (35.7, 43.2)	<.001
≤40	450 (53.3)	88 (72.1)	362 (50.1)	<.001
Globulin	25.1 (22.2, 29.1)	26.5 (23.2, 29.6)	24.7 (22, 28.9)	.004
≥30	166 (19.6)	30 (24.6)	136 (18.8)	.137
Glucose (mmol/L)	5.59 (4.89, 6.99)	6.3 (5.1, 7.9)	5.5 (4.8, 6.9)	.005
≥6	372 (44)	64 (52.5)	308 (42.6)	.04
APTT (s)	27.8 (25.6, 30.9)	28.7 (25.9, 32.8)	27.7 (25.5, 30.5)	.033
≥37	43 (5.1)	14 (11.5)	29 (4)	.001
PT (s)	12 (11.3, 12.8)	12.5 (11.8, 13.4)	12 (11.2, 12.7)	<.001
≥14	83 (9.8)	24 (19.7)	59 (8.2)	<.001
INR	1.03 (0.96, 1.09)	1.05 (0.99, 1.14)	1.02 (0.95, 1.08)	<.001
≥1.2	40 (4.7)	11 (9)	29 (4)	.02
Fibrinogen (g/L)	3.56 (2.61, 4.82)	4.32 (3.29, 6.03)	3.49 (2.56, 4.69)	<.001
≥4	268 (31.7)	62 (50.8)	206 (28.5)	<.001
D‐dimer (mg/L)	0.58 (0.28, 1.48)	1.16 (0.51, 4.4)	0.53 (0.26, 1.21)	<.001
≥0.5	366 (43.3)	82 (67.2)	284 (39.3)	<.001
C‐reactive protein (mg/L)	27.3 (7.8, 64.3)	57.9 (16.8, 125.2)	24.1 (6.7, 55.48)	<.001
≥5	289 (34.2)	59 (48.4)	230 (31.8)	<.001
Procalcitonin (μg/L)	0.05 (0.03, 0.1)	0.11 (0.05, 0.29)	0.05 (0.03, 0.08)	<.001
≥0.5	27 (3.2)	14 (11.5)	13 (1.8)	<.001
Interleukin‐6	5.77 (3.86, 11.37)	7.37 (2.57, 39.83)	5.72 (3.99, 10.64)	.77
CD3+ cell counts (cell/μl)	750 (503, 1102)	475 (282, 659)	841 (551, 1149)	<.001
≤955	279 (33)	57 (46.7)	222 (30.7)	.001
CD4+ cell counts (cell/μl)	457 (278, 673)	300 (192, 449)	493 (305, 712)	<.001
≤450	201 (23.8)	44 (36.1)	157 (21.7)	.001
CD8+ cell counts (cell/μl)	263 (158, 395)	141 (74, 220)	288 (174, 416)	<.001
≤320	256 (30.3)	52 (42.6)	204 (28.2)	.001

*Note*: Data are shown as median with interquartile range (IQR) for continuous variables or number with percentage for categorical variables.

Abbreviations: ALT, alanine aminotransferase; APTT, activated partial thromboplastin time; AST, aspartate aminotransferase; COPD, chronic obstructive pulmonary disease; CT, computed tomography; eGFR, estimated glomerular filtration rate; INR, international normalized ratio; *n*, numbers; PT, prothrombin time.

### 3.2 Risk factors and risk score

The factors with *p* < .10 in Table 1 were added into the logistic regression model analysis. In univariate and multivariate analysis, continuous variables were converted to categorical variables. Finally, 10 independent risk factors associated with cardiovascular complications were identified: male (OR: 1.84; 95% CI: 1.18, 2.85), age ≥ 60 years old (OR: 2.01; 95% CI: 1.3, 3.2), cough (OR: 1.86; 95% CI: 1.16, 3), chronic heart disease (OR: 2.3; 95% CI: 1.19, 4.46), lymphocyte count ≤1.1 × 10^9^/L at admission (OR: 1.60; 95% CI: 1.03, 2.47), blood urea nitrogen ≥7 mmol/L at admission (OR: 2.14; 95% CI: 1.27, 3.62), eGFR ≤ 90 ml/min/1.73 m^2^ at admission (OR: 2.08; 95% CI: 1.13, 3.83), activated partial thromboplastin time (APTT) ≥ 37 s (OR: 3.07; 95% CI: 1.37, 6.86), D‐dimer ≥ 0.5 mg/L (OR: 2.12; 95% CI: 1.33, 3.36), and procalcitonin ≥0.5 μg/L (OR: 3.58; 95% CI: 1.40, 9.14). The details and corresponding score for each risk factor are showed in Table 2.

**Table 2:** Independent risk factors associated with cardiovascular complications in multivariate analysis and corresponding risk score in training set

Risk factors	Multivariate OR (95% CI)	*p* value	Score
Sex (male)	1.84 (1.18, 2.85)	.007	2
Age (years, ≥60)	2.01 (1.3, 3.2)	.002	2
Cough	1.86 (1.16, 3)	.01	2
Chronic heart disease	2.3 (1.19, 4.46)	.01	2
Lymphocyte count (×10^9^/L, ≤1.1)	1.60 (1.03, 2.47)	.04	2
Blood urea nitrogen (mmol/L, ≥7)	2.14 (1.27, 3.62)	.004	2
eGFR (ml/min/1.7 m^2^, ≤90)	2.08 (1.13, 3.83)	.02	2
APTT (s, ≥37)	3.07 (1.37, 6.86)	.006	3
D‐dimer (mg/L, ≥0.5)	2.12 (1.33, 3.36)	.001	2
Procalcitonin (μg/L, ≥0.5)	3.58 (1.40, 9.14)	.008	4

Abbreviations: APTT, activated partial thromboplastin time; CI, confidence interval; eGFR, estimated glomerular filtration rate; OR, odds ratio.

**Figure 2:**
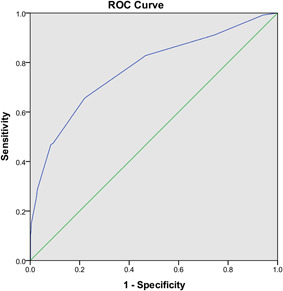
Receiver operating characteristic (ROC) curve for prediction of cardiovascular complications in COVID‐19 patients. Area under the curve was 0.773 (95% confidence interval: 0.723, 0.822; *p* < .01). COVID‐19, coronavirus disease 2019

As a result, the total risk score for each patient varied from 0 to 23 points. Finally, an optimal cut‐off value of 7.5 (specificity: 0.656, sensitivity: 0.780) was identified to predict cardiovascular complications according to ROC curve and AUC was 0.773 (95% CI: 0.723, 0.822; *p* < .01; Figure 2).

### 3.3 Validation and survival analysis

In the testing set, 62 (17.1%) patients were found to have cardiovascular complications. The accuracy of the risk score in the testing set (AUC: 0.756; 95% CI: 0.690, 0.822) was similar to that in the training set. Furthermore, the AUC of the risk score in overall patients (training set plus testing set) was 0.766 (95% CI: 0.726, 0.806). The optimal cut‐off value is also 7.5 (specificity: 0.620; sensitivity: 0.785). The results are summarized in Table 3. Generally, our results were relatively stable and reliable and the novel risk score had generalizability, to some degree.

**Table 3:** Performance of novel risk score

Data set	Patients with cardiovascular complications/overall (%)	AUC (95% CI)	Optimal cut‐off value	Sensitivity	Specificity
Training set	122/845 (14.4)	0.773 (0.723, 0.822)	7.5	0.656	0.780
Testing set	62/362 (17.1)	0.756 (0.690, 0.822)	5.50	0.823	0.573
Training set plus testing set	184/1207 (15.2)	0.766 (0.726, 0.806)	7.50	0.620	0.785

Abbreviations: AUC, area under the curve; CI, confidence interval.

**Figure 3:**
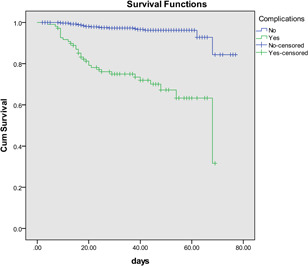
Kaplan–Meier curves according to cardiovascular complications in COVID‐19 patients (log‐rank test: *p* < .001). COVID‐19, coronavirus disease 2019

In addition, cardiovascular complications were significantly associated with poorer survival (log‐rank test: *p* < .001) in the Kaplan–Meier curves, which showed that cardiovascular complications had considerable adverse impacts on the prognosis of COVID‐19 patients (Figure 3).

## 4 DISCUSSION

To our knowledge, this is the first predictive tool used for predicting cardiovascular complications among COVID‐19 patients at admission to hospital. Ten independent risk factors at admission were identified and the total score varied from 0 to 23 points for each patient. The risk score has also been validated. A higher total score is correlated to increased risk of cardiovascular complications which might lead to significantly poor prognosis of COVID‐19 patients. Therefore, early prediction of cardiovascular complications is important and necessary.

Wei et al.^15^ have ever conducted a similar study and found age, pre‐existing cardiovascular disease, eGFR, and procalcitonin were associated with acute myocardial injury (defined as an hs‐TnT [high‐sensitivity troponin T] value >14 pg/ml) in COVID‐19 patients. Meanwhile, acute myocardial injury was more correlated to severe/critical, admission to ICU, mechanical ventilation, and death. In the current study, more baseline variables were included. Furthermore, the primary outcome of composite cardiovascular events was adopted because they should all be considered as important cardiovascular complications of COVID‐19.^16^ It is believed that a risk score might enable more accurate identification and stratification than a single predictor for COVID‐19 patients. Therefore, based on independent risk factors, we developed a novel, practical predictive risk score. As a bedside tool, this score system comprehensively consists of factors of demographic characteristics, symptoms, comorbidities, and laboratory examinations.

According to previous reports, cardiovascular complications were common clinical manifestations and could increase the health burden of SARS and influenza in the last decades.^17^, ^18^ The potential mechanisms for cardiovascular complications during SARS‐CoV‐2 infection has not been thoroughly explained. Possible mechanisms include direct virus‐mediated cardiotoxicity, hypoxia‐related injury, immune‐mediated cytokine storm and systemic inflammation, and so forth.^19^ Infection of the pericardium causing massive edema and myocardial fibrosis or scars have also been put forward in a recent review.^20^ Besides, increased heart burden and cardiopulmonary dysfunction caused by SARS‐CoV‐2 infection might be responsible for the myocardial ischemia, worsening HF, and new arrhythmia.

Previous studies have demonstrated that myocardial injury occurred in about 10% of patients with COVID‐19.^4^, ^9^ In the current study, composite cardiovascular complications were reported in 15.2% of COVID‐19 patients. We included 917 (74%) patients from Wuhan city, a high‐risk and high‐prevalence area, and 323 (26%) patients from Sichuan province which is a low‐risk district. Furthermore, these patients were randomly assigned to either the training set or the testing set in an attempt to draw a relatively comprehensive and fair conclusion. However, it must be noted that incidence and types of cardiovascular complications might be associated with severities of illness and population characteristics. Future studies are warranted to verify our conclusions.

In the risk score, some factors have been widely confirmed to be connected with cardiovascular events, including male, older age, chronic heart disease, prolonged APTT, elevated D‐dimer, and so on. Their predictive values for cardiovascular complications have also been demonstrated in community‐acquired pneumonia.^12^ However, some arisen independent risk factors in multivariable analysis were unexpected or uncommon, which should be treated cautiously. Irwin^21^ has ever conducted a literature review and found cough, an effective means of clearing the airways, could also cause a variety of complications, including cardiovascular complications. However, the impact of cough on patients still remains to be clarified. Decreased lymphocyte count is common in COVID‐19 patients according to previous studies.^4^, ^9^ Systemic inflammatory response and immunocompromised status might be responsible for it. One previous study also reported that the number of helper T cells and suppressor T cells both significantly decreased, and were more impaired in severe COVID‐19 cases. The authors concluded that SARS‐CoV‐2 might mainly act on lymphocytes, especially T lymphocytes.^22^ It is also reported that lymphopenia plays a role in accelerated atherosclerosis and increased incidence of cardiovascular events.^23^ The association between blood urea nitrogen or eGFR and cardiovascular complications in COVID‐19 is still unclear now. However, our results are consistent with a previous study of influenza. Nin et al.^24^ have revealed that patients with AKI presented more cardiovascular dysfunction compared with those without AKI in 2009 influenza A (H1N1) viral pneumonia. Procalcitonin often has high accuracy for the diagnosis of bacterial infections in clinical practice. It is understandable that patients with cardiovascular complications were more likely to be severe cases and coinfected with bacteria due to immunosuppression. Additionally, in one previous population‐based prospective study, procalcitonin was also found to be correlated to several of the established cardiovascular risk factors (C‐reactive protein, hypertension, renal function, etc.) and positively associated with cardiovascular events and cardiovascular death.^25^

We found that some risk factors in the current study were also applied in previous similar prediction models for critical illness, admission to ICU, or mortality of COVID‐19 patients. For instance, Gong et al.^26^ developed a nomogram in which older age and blood urea nitrogen were associated with severe COVID‐19. In another clinical prediction model for in‐hospital mortality of COVID‐19 patients, age, history of heart disease, lymphocyte count, D‐dimer, and eGFR were used.^27^ It has to be acknowledged that the AUC of the current risk score is inferior to those of the above prediction models, which varied from 0.8 to 0.9. We speculated that some baseline data were missing due to a retrospective study design, which might have compromised the discriminatory power of this risk score. Then, it is possible that the endpoint of cardiovascular complications was more heterogeneous than that of severe illness or death. And the severities of various cardiovascular complications were not identical among included patients. However, our study has a larger sample size (over 1200 patients). Clear and widely accepted definitions were adopted for all included patients and the diagnosis of cardiovascular complications was checked by different researchers to guarantee the accuracy of our conclusions.

Given the lack of specific antiviral agents for SARS‐CoV‐2 and significant adverse effects of cardiovascular complications on the prognosis of patients, early identification of COVID‐19 patients at high risk of cardiovascular complications, timely intervention, and protection of target organ are important and essential. In the current study, all risk factors are easy to obtain at admission to hospital and this risk score has been well developed and validated with promising predictive capacity. It might help clinicians make optimal treatment decisions for patients who are prone to develop cardiovascular complications, and help researchers explore more detailed systemic damage and pathophysiological mechanisms of COVID‐19 in the future.

There are several limitations to our study. First, it was a retrospective observational study with potential unavoidable selection bias. Second, the sample size was relatively moderate, and the number of patients was not equal between groups with or without cardiovascular complications. Third, the drugs and therapies before admission might have considerable impacts on our results. Then, we only recorded cardiovascular complications before discharge without follow‐up. The long‐term damage of SARS‐CoV‐2 to cardiovascular systems and related risk factors remain to be explored. Further well‐designed, multicenter, long‐term studies with better comparability are warranted to clarify the characteristics of these risk factors and verify our conclusions.

## 5 CONCLUSIONS

We developed and validated a novel risk score, which is based on 10 risk factors at admission: male, age ≥ 60 years, cough, chronic heart disease, lymphocyte count ≤1.1 × 10^9^/L, blood urea nitrogen ≥7 mmol/L, eGFR ≤ 90 ml/min/1.73 m^2^, APTT ≥ 37 s, D‐dimer ≥ 0.5 mg/L and procalcitonin ≥0.5 μg/L. This risk score has a promising predictive capacity for cardiovascular complications which could significantly impair the prognosis of COVID‐19 patients. Our conclusions need to be further confirmed in further studies.

## CONFLICT OF INTERESTS

The authors declare that there are no conflict of interests.

## AUTHOR CONTRIBUTIONS

Dong Huang, Huan Yang, He Yu, Ting Wang, Rong Yao, and Zongan Liang conceived the idea, designed, and supervised the study, drafted the manuscript, and had full access to all of the data and took responsibility for the integrity of the data. Dong Huang and Huan Yang collected data. Dong Huang and Huan Yang analyzed data and performed the statistical analysis. All of the authors reviewed and approved the final version of the manuscript.

## References

[1] Zhu N , Zhang D , Wang W , et al. A novel coronavirus from patients with pneumonia in China, 2019. N Engl J Med. 2020;382(8):727‐733. 10.1056/NEJMoa2001017 31978945 PMC7092803

[2] Li Q , Guan X , Wu P , et al. Early transmission dynamics in Wuhan, China, of novel coronavirus‐infected pneumonia. N Engl J Med. 2020;382(13):1199‐1207. 10.1056/NEJMoa2001316 31995857 PMC7121484

[3] COVID‐19 Map—Johns Hopkins Coronavirus Resource Center. https://coronavirus.jhu.edu/map.html. Accessed July 30, 2020.

[4] Huang C , Wang Y , Li X , et al. Clinical features of patients infected with 2019 novel coronavirus in Wuhan, China. Lancet. 2020;395(10223):497‐506. 10.1016/S0140-6736(20)30183-5 31986264 PMC7159299

[5] Driggin E , Madhavan MV , Bikdeli B , et al. Cardiovascular considerations for patients, health care workers, and health systems during the COVID‐19 pandemic. J Am Coll Cardiol. 2020;75(18):2352‐2371. 10.1016/j.jacc.2020.03.031 32201335 PMC7198856

[6] Wu Z , McGoogan JM . Characteristics of and important lessons from the coronavirus disease 2019 (COVID‐19) outbreak in China: summary of a report of 72314 cases from the Chinese Center for Disease Control and Prevention. JAMA. 2020;323:1239 10.1001/jama.2020.2648 32091533

[7] Ruan Q , Yang K , Wang W , Jiang L , Song J . Clinical predictors of mortality due to COVID‐19 based on an analysis of data of 150 patients from Wuhan, China. Intensive Care Med. 2020;46(5):846‐848. 10.1007/s00134-020-05991-x 32125452 PMC7080116

[8] Zhou F , Yu T , Du R , et al. Clinical course and risk factors for mortality of adult inpatients with COVID‐19 in Wuhan, China: a retrospective cohort study. Lancet. 2020;395(10229):1054‐1062. 10.1016/S0140-6736(20)30566-3 32171076 PMC7270627

[9] Wang D , Hu B , Hu C , et al. Clinical characteristics of 138 hospitalized patients with 2019 novel coronavirus‐infected pneumonia in Wuhan, China. JAMA. 2020;323(11):1061‐1069. 10.1001/jama.2020.1585 32031570 PMC7042881

[10] World Health Organization . Clinical management of severe acute respiratory infection when COVID‐19 is suspected: interim guidance; 13 March 2020. https://www.who.int/publications-detail/clinical-management-of-severe-acute-respiratory-infection-when-novel-coronavirus-(ncov)-infection-is-suspected. Accessed June 1, 2020.

[11] Menéndez R , Méndez R , Aldás I , et al. Community‐acquired pneumonia patients at risk for early and long‐term cardiovascular events are identified by cardiac biomarkers. Chest. 2019;156(6):1080‐1091. 10.1016/j.chest.2019.06.040 31381883

[12] Violi F , Cangemi R , Falcone M , et al. Cardiovascular complications and short‐term mortality risk in community‐acquired pneumonia. Clin Infect Dis. 2017;64(11):1486‐1493. 10.1093/cid/cix164 28205683

[13] Thygesen K , Alpert JS , Jaffe AS , et al. Fourth Universal Definition of Myocardial Infarction (2018). Circulation. 2018;138(20):e618‐e651. 10.1161/CIR.0000000000000617 30571511

[14] Yancy CW , Jessup M , Bozkurt B , et al. 2017 ACC/AHA/HFSA focused update of the 2013 ACCF/AHA guideline for the management of heart failure: a report of the American College of Cardiology/American Heart Association Task Force on Clinical Practice Guidelines and the Heart Failure Society of America. Circulation. 2017;136(6):e137‐e161. 10.1161/CIR.0000000000000509 28455343

[15] Wei JF , Huang FY , Xiong TY , et al. Acute myocardial injury is common in patients with covid‐19 and impairs their prognosis. Heart. 2020;106:1154‐1159. 10.1136/heartjnl-2020-317007 32354798 PMC7398466

[16] Long B , Brady WJ , Koyfman A , Gottlieb M . Cardiovascular complications in COVID‐19. Am J Emerg Med. 2020;38:1504‐1507. 10.1016/j.ajem.2020.04.048 32317203 PMC7165109

[17] Yu CM , Wong RS , Wu EB , et al. Cardiovascular complications of severe acute respiratory syndrome. Postgrad Med J. 2006;82(964):140‐144. 10.1136/pgmj.2005.037515 16461478 PMC2596695

[18] Estabragh ZR , Mamas MA . The cardiovascular manifestations of influenza: a systematic review. Int J Cardiol. 2013;167(6):2397‐2403. 10.1016/j.ijcard.2013.01.274 23474244

[19] Zhu H , Rhee JW , Cheng P , et al. Cardiovascular complications in patients with COVID‐19: consequences of viral toxicities and host immune response. Curr Cardiol Rep. 2020;22(5):32 10.1007/s11886-020-01292-3 32318865 PMC7171437

[20] Siripanthong B , Nazarian S , Muser D , et al. Recognizing COVID‐19‐related myocarditis: the possible pathophysiology and proposed guideline for diagnosis and management. Heart Rhythm. 2020;17:1463‐1471. 10.1016/j.hrthm.2020.05.001 32387246 PMC7199677

[21] Irwin RS . Complications of cough: ACCP evidence‐based clinical practice guidelines. Chest. 2006;129(1 suppl):54S‐58S. 10.1378/chest.129.1_suppl.54S 16428692

[22] Qin C , Zhou L , Hu Z , et al. Dysregulation of immune response in patients with COVID‐19 in Wuhan, China. Clin Infect Dis. 2020;71:762‐768. 10.1093/cid/ciaa248 32161940 PMC7108125

[23] Nunez J , Minana G , Bodi V , et al. Low lymphocyte count and cardiovascular diseases. Curr Med Chem. 2011;18(21):3226‐3233. 10.2174/092986711796391633 21671854

[24] Nin N , Lorente JA , Soto L , et al. Acute kidney injury in critically ill patients with 2009 influenza A (H1N1) viral pneumonia: an observational study. Intensive Care Med. 2011;37(5):768‐774. 10.1007/s00134-011-2167-7 21394630 PMC7095219

[25] Schiopu A , Hedblad B , Engström G , Struck J , Morgenthaler NG , Melander O . Plasma procalcitonin and the risk of cardiovascular events and death: a prospective population‐based study. J Intern Med. 2012;272(5):484‐491. 10.1111/j.1365-2796.2012.02548.x 22530956

[26] Gong J , Ou J , Qiu X , et al. A tool to early predict severe corona virus disease 2019 (COVID‐19): a multicenter study using the risk nomogram in Wuhan and Guangdong, China. Clin Infect Dis. 2020;71:833‐840. 10.1093/cid/ciaa443 32296824 PMC7184338

[27] Wang K , Zuo P , Liu Y , et al. Clinical and laboratory predictors of in‐hospital mortality in patients with COVID‐19: a cohort study in Wuhan, China [published online ahead of print May 3, 2020]. Clin Infect Dis. 2020:ciaa538 10.1093/cid/ciaa538 32361723 PMC7197616

